# RNAi-mediated mortality of the whitefly through transgenic expression of double-stranded RNA homologous to acetylcholinesterase and ecdysone receptor in tobacco plants

**DOI:** 10.1038/srep38469

**Published:** 2016-12-08

**Authors:** Hassan Jamil Malik, Amir Raza, Imran Amin, Jodi A. Scheffler, Brian E. Scheffler, Judith K. Brown, Shahid Mansoor

**Affiliations:** 1 Molecular Virology and Gene Silencing Laboratory, Agricultural Biotechnology Division, National Institute for Biotechnology and Genetic Engineering (NIBGE), Jhang Road, PO Box #577, Faisalabad, Pakistan; 2 Pakistan Institute of Engineering and Applied Sciences (PIEAS), Islamabad, Pakistan; 3 USDA-ARS, Crop Genetics Research Unit, 141 Experiment Station Rd, Stoneville, MS 38776, USA; 4 USDA-ARS, Genomics and Bioinformatics Research Unit, 141 Experiment Station Rd, Stoneville, MS 38776, USA; 5 School of Plant Sciences, The University of Arizona, Tucson, AZ 85721, USA

## Abstract

The whitefly *Bemisia tabaci* (Genn.) is a pest and vector of plant viruses to crop and ornamental plants worldwide. Using RNA interference (RNAi) to down regulate whitefly genes by expressing their homologous double stranded RNAs in plants has great potential for management of whiteflies to reduce plant virus disease spread. Using a *Tobacco rattle virus*-derived plasmid for *in planta* transient expression of double stranded RNA (dsRNA) homologous to the *acetylcholinesterase (AChE*) and *ecdysone receptor (EcR*) genes of *B. tabaci*, resulted in significant adult whitefly mortality. *Nicotiana tabacum* L. plants expressing dsRNA homologous to *B. tabaci AChE* and *EcR* were constructed by fusing sequences derived from both genes. Mortality of adult whiteflies exposed to dsRNA by feeding on *N. tabacum* plants, compared to non-dsRNA expressing plants, recorded at 24-hr intervals post-ingestion for three days, was >90% and 10%, respectively. Analysis of gene expression by real time quantitative PCR indicated that whitefly mortality was attributable to the down-regulation of both target genes by RNAi. Results indicated that knock down of whitefly genes involved in neuronal transmission and transcriptional activation of developmental genes, has potential as a bio-pesticide to reduce whitefly population size and thereby decrease virus spread.

Management approaches to reduce the negative effects caused by insect damage to agricultural crops have undergone a considerable shift as the result of a number of recent technological advancements in pest control. An important insect of cotton such as *Helicoverpa armigera* has been controlled worldwide by expressing an insecticidal protein *in planta* obtained from *Bacillus thuringiensis(Bt)*^1^. Transgenic crops that offer protection from insect pests have aided the agriculture sector by reducing damage to crops resulting from insect damage^2^. However, there are limitations to the Bt technology including off-target effects to beneficial insects such as natural/biological predators of harmful pests and pollinators^3^ and its ineffectiveness against sap-sucking insects belonging to the order Hemiptera^4^. The great potential for using transgenic plants to control phloem-feeding insects belonging to the order, Hemiptera has not been realized, in part, owing to the need to identify and optimize strategies that target and undermine key insect receptors.

RNA interference (RNAi) technology is based on the expression of double stranded RNA (dsRNA) that shares nearly 100% sequence homology with a desired target gene for optimal silencing^5^ and has been widely used in functional genomics studies carried out on insects^6^. RNAi is a natural mechanism present in eukaryotes that serves as a defense mechanism against viruses and is also involved in the regulation of gene expression depending upon internal and external environmental conditions^7^. This phenomenon in animals was first demonstrated in *Caenorhabditis elegans* by silencing of *unc-22* gene in a specific and systemic manner^8^. Primarily, the mechanism of RNAi mediated gene silencing is based on the exogenous production of short interfering RNAs/microRNAs (siRNAs/miRNAs) by an organism to control the expression of genes. The process can also be triggered in insect cells either by expression of dsRNA or by dsRNA imported into the cells by a transporter protein such as systemic RNA interference defective-1(SID-1)^9^. The long dsRNA is cleaved by an RNaseIII type nuclease known as Dicer into siRNAs ranging in size from 21–25 bp with 2 nucleotide 3′ overhangs. The siRNAs are recruited by the RNA-induced silencing complex (RISC), a multi-protein complex where one strand is cleaved and degraded, referred to as the passenger, while the other serves as the guide strand. The passenger strand is targeted for degradation upon cleavage, and guide strand integration directs the RISC complex to bind the specific target messenger RNA. When RISC detects the target mRNA with the aid of the guide strand, it attaches to the target, which is degraded by the RNase component of the RISC complex, known as, Argonaute^10^. A number of different applications for RNAi have been reported, including its use to cause mortality in insects^11^. Double-stranded RNA has been shown to be persistent in the plasma hemolymph of *Blattella germanica*, making it lucrative for experimental RNAi studies^12^. Injection of dsRNA into the pea aphid, *Acyrthosiphon pisum,* resulted in the silencing of the genes *Ap-crt* and *Ap-cath-L* that encode calreticulin and cathepsin-L, respectively^13^. RNAi-mediated knockdown of midgut genes (*Nlsid-1* and *Nlaub*) of *Nilaparvata lugens* demonstrated a promising role of RNAi for controlling phytophagous insects in the field^14^. Suppression of two chitin synthase genes (*AgCHS1* and *AgCHS2*) present in African malaria mosquito (*An. gambiae*) larvae reduced larval chitin content, which resulted in increased vulnerability to the insecticide, diflubenzuron^15^.

The ability to express dsRNA in transgenic plants for RNAi-mediated control of insects offers an effective way to control pests of economic importance, as plants retain RNAi machinery which imparts in them a natural ability for cross-kingdom gene silencing^16^. Many reports have demonstrated the potential for transgenic plant-mediated pest control by expressing dsRNA homologous to genes essential for insect survival^11^. For example, transgenic *Arabidopsis thaliana* targeting the *Rack1* gene and transgenic *Nicotiana benthamiana* targeting the *MPC002* gene of *Myzus persicae* have been reported to adversely affect aphid life span^17^. Silencing of the *CYP6AE14c* gene impaired *Helicoverpa armigera* larval resistance to gossypol in *Gossypium hirsutum* L.^18^ and Western corn rootworm was reported to be effectively controlled by transgenic *Zea mays* L. expressing dsRNA against the *V-ATPase-A* gene^19^.

Among phloem-feeding insect pests of agricultural importance, the *Bemisia tabaci* (Genn.) sibling species group is one of the most damaging, causing losses in agronomic and horticultural crops, nearly worldwide^20^. Collectively, *B. tabaci* has a broad host range consisting of approximately 500 plant species, worldwide. It causes yield losses by feeding damage, and through the transmission of plant viruses that undermine plant growth and productivity^21^. Outbreaks caused by cotton leaf curl disease (CLCuD) have resulted in reduced cotton and vegetable crop production, with losses as high as 100%^22^. A greater understanding of the status and dynamics of *B. tabaci* in Pakistan cropping systems is needed to more effectively combat CLCuD. Previous studies have identified *B. tabaci* haplotypes and biotypes^23^ from Pakistan, including the exotic, invasive MEAM I haplotype (also known as the B biotype) that groups in the North Africa-Mediterranean-Middle East (NA-MED-ME) clade (also, Middle East Asia Minor 1) that was present in only Sindh Province, and two endemic cryptic species that group in the Asia 1 and Asia II 1 clades. The Asia 1 cryptic species was identified in Punjab and Sindh Province, whereas, Asia II 1 was present only in Punjab Province^24^,^25^,^26^. In a recent study that analyzed 593 whitefly collections from 255 locations of Pakistan, the presence of the aforementioned two cryptic species, was confirmed, including Asia II-5 from cotton and Asia II-7 from cotton and non-cotton hosts, and an additional previously uncharacterized species was reported from cotton plants, referred to as ‘Pakistan’^25^.

The increased invasiveness of certain *B. tabaci*, has been attributed to higher fecundity, and to the development of resistance to organophosphate, pyrethroid and neonicotinoid insecticides^27^,^28^,^29^,^30^. Whitefly management has therefore become of great concern in agricultural production systems. Whitefly mortality has been achieved by expressing dsRNA in transgenic tobacco plants targeting the *V-ATPase A* gene, resulting in a significant reduction in the whitefly population size within 8 days, post-exposure^31^. Another recent report has demonstrated the potential of RNAi technology for *Bemisia* control by down-regulating midgut specific genes involved in osmoregulation^32^.

In this study, we have targeted using RNAi two genes involved in signaling pathways important for whitefly development and neural transmission, hypothesizing that interference with these essential pathways could be lethal. *Acetylcholinesterase (AChE*) is involved in hydrolysis of the neurotransmitter acetylcholine into its acetyl-CoA and acetate components, thereby clearing the residual neurotransmitter molecules from the synaptic cleft that regulate normal behavior. Blockage of *AChE* leads to increased acetylcholine levels, causing the continuous stimulation of muscles and glands, and resulting in muscular dysfunction, paralysis, and death. *AChE* also has a vital role in neurite outgrowth and synapse formation^33^,^34^. For example, RNAi mediated silencing of *AChE* delivered by dsRNAs ingested by *Helicoverpa armigera* and *Blattella germanica,* underscores the potency of this target for gene knock downs in insects, much as has been previously achieved with commercial insecticides^35^,^36^. The *ecdysone receptor* gene*, EcR,* and second target examined in this study, is involved in the steroid signaling pathway whose activation induces a cascade of ecdysone responsive genes that are critical for insect development, and is also important for molecular structure and function of a number of gene products. In recent studies, dsRNA expression to down-regulate *EcR* function in adult *Drosophila melanogaster,* demonstrated that *EcR* decreased expression can perturb reproduction, courtship behavior, and stress resistance^37^,^38^, as well as adversely affecting survival and sex-mediated signaling in adults^39^.

Here, tobacco plants transient expression of dsRNA homologous to sequences of the *B. tabaci acetylcholinesterase* and *ecdysone receptor* gene, respectively, were tested separately using *Tobacco rattle virus* (TRV)-induced gene silencing (VIGS) vector (TRV-VIGS) for dsRNA expression in *N. tabacum*. The TRV-VIGS consists of the modified viral genomic component, TRV RNA1 that encodes viral movement, and TRV RNA2, which is involved host plant gene silencing, a system first designed to study transiently functional genomics of viral-plant interactions^40^, without requiring stable plant transformation. Results indicate that transiently, as well as transgenically expressed dsRNA induced RNAi, resulted in whitefly mortality in adult whitefly, post-dsRNA ingestion from tobacco plants.

## Results

### DNA sequencing and analysis of *AChE* and *EcR* sequences for dsRNA design

**Figure 1:**
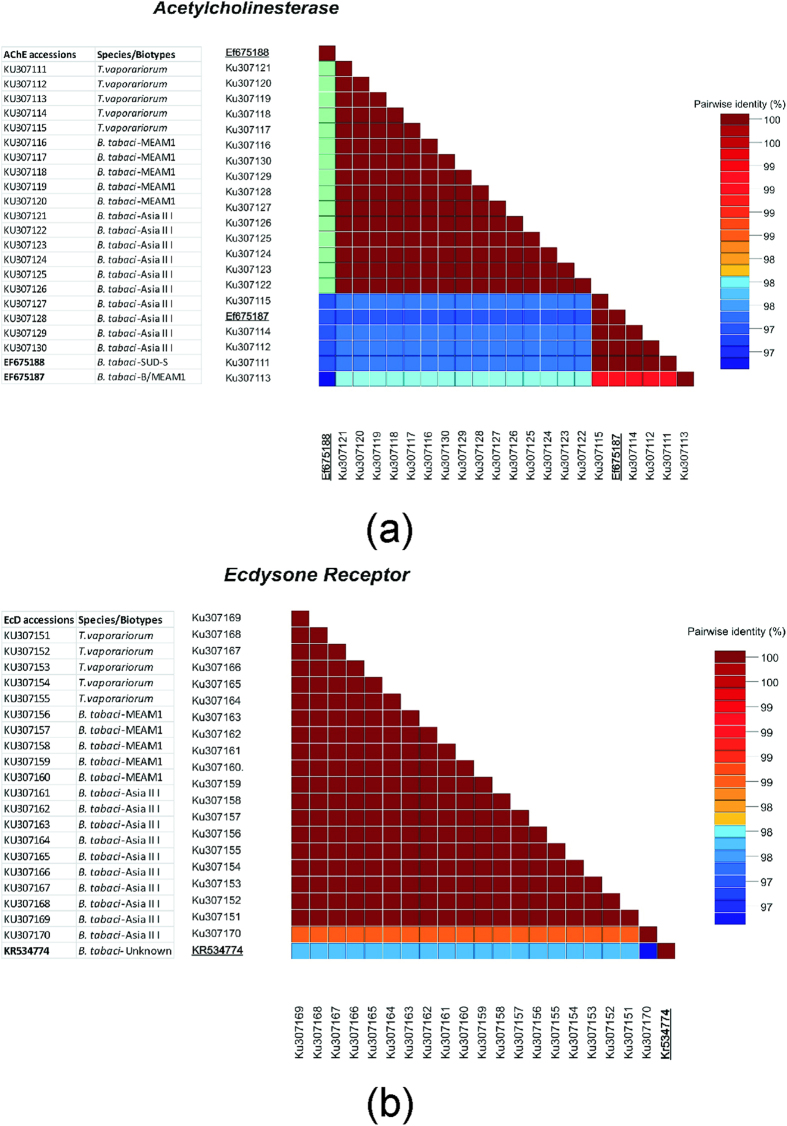
Distance matrix demonstrating high conservation of target genes *AChE* (**a**) and *EcR* (**b**) among different whitefly haplotypes found in different regions of Pakistan and the greenhouse whitefly *Trialeurodes vaporariorum*.

Twenty sequences of 200 bp were obtained by PCR for 20 different field collections of *B. tabaci* from Pakistan, and have been deposited in the GenBank database as Accession numbers KU307111 – KU307130 for *AChE* and KU307151 – KU307170 (sequences not released) for *EcR*. Multiple sequence alignment of the *AChE* and *EcR* from Pakistan, was carried out with MegAlign software using the ClustalW method. Consensus sequences were generated from the alignments and the results revealed that both the sequences have +60 bp nucleotides conserved among *T. vaporariorum* and two *B. tabaci*, MEAM I species (representative *mtCOI* accessions: KX675904 – KX675908) and Asia II (representative *mtCOI* accessions: KX675909 – KX675918) present in Pakistan (Table S1 and Figure S1). The sequence distance matrix generated using SDT v1.2 showed that *AChE* sequences were 100% conserved among the Pakistan-endemic *B. tabaci* species (Fig. 1a). The *EcR* sequences also demonstrated 100% conservation among *T. vaporariorum* and *B. tabaci* (Fig. 1b). One isolate of Asia II I showed 98% similarity with the rest of the sequences in *EcR* clade.

### Whitefly bioassay on plants transiently expressing dsRNA

**Figure 2:**
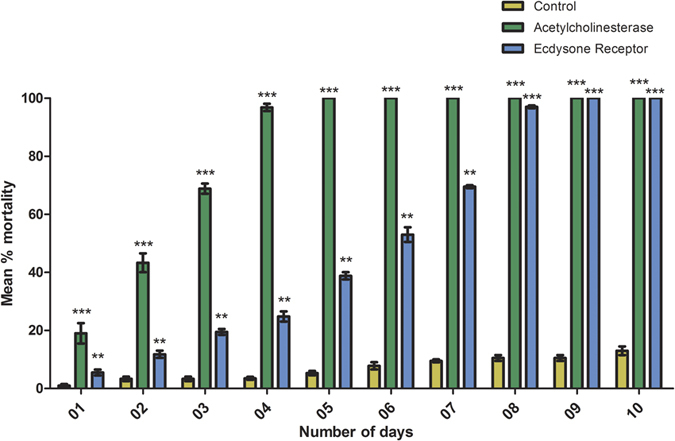
Statistical analysis of whitefly mortality for VIGS-TRV bioassays. Significantly higher mortality compared to the control showed that the target genes were highly efficacious for the control of whitefly.

The target genes were transiently expressed in *N. tabacum* plants using the TRV-VIGS vector system because of its usefulness as a VIGS vector. The experiment was performed to assess the efficacy of dsRNA expression against whitefly target genes before proceeding to stably transforming plants. Significantly higher adult whitefly mortality, at 40%, was recorded 48 hrs post-plant exposure, and reached 98% after four days for tobacco plants transiently expressing dsRNA homologous to *AChE*. And, greater than 50% mortality was observed after six days of whiteflies feeding on tobacco plants transiently expressing dsRNA against whitefly *EcR*. Mortality was 100% by nine days post-plant exposure to *EcR* dsRNA transiently expressed in tobacco plants. Statistical analysis of mortality data (Fig. 2) demonstrated significantly higher mortality in whiteflies exposed to dsRNA of both target genes, compared to the negative controls, for which only 13% mortality was recorded after ten days. A *p*-value < 0.05 indicated that results were highly significant.

### Transgenic tobacco plants expressing dsRNA

To target the genes of whiteflies having an essential role in cellular signaling through transgenic tobacco, a construct was developed in a pJIT163 vector in such a way that partial fragments of *AChE* and *EcR* were simultaneously expressing under the control of a *Figwort mosaic virus* (FMV) promoter. An intron was added to separate the sense and anti-sense fragments to facilitate configuration of a hairpin mRNA upon transcription. Total DNA was isolated from the leaf tissue of the plants. Confirmation of gene insertion into the plants was established by PCR using gene specific as well as *nptII* gene specific primer pairs.

### Expression of *AChE* and *EcR* dsRNA cassettes in transgenic tobacco plants

**Figure 3:**
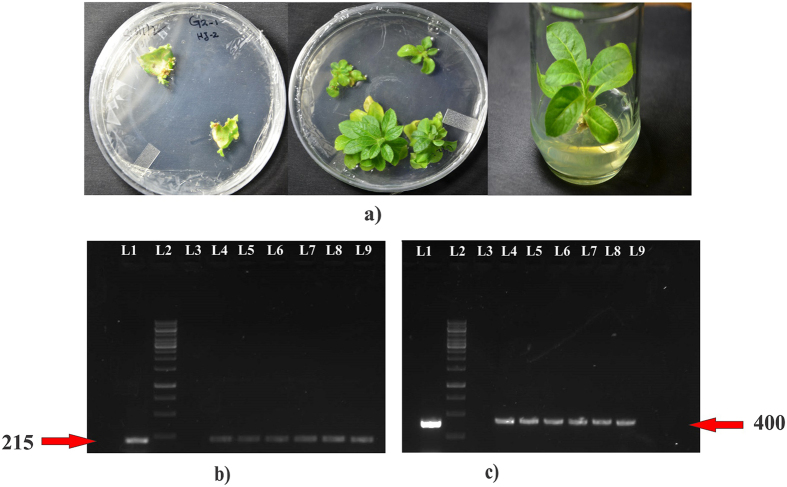
Plant transformation and transgene analysis (**a**) selection of the tobacco transformants following Agrobacterium-mediated transformation with the G2 cassette and kanamycin media selection (**b**) Confirmation of the nptII gene for the kanamycin resistance in the transgenic lines by PCR-amplification of product size 215 bp. Loading arrangement of the samples in 1% agarose gel is, Lane 1 = positive control, Lane 2 = 1 kb DNA ladder, Lane 3 = negative control, Lane 4 –6 = transgenic line G2.H2-4, and Lane 7–9 = transgenic line G2.H3-5 (**c**) confirmation that construct inserted into the nuclear genome of the tobacco plants was carried out by PCR-amplification of target gene-specific primers of 400 bp product size. Loading arrangement of the samples in 1% agarose gel is as, Lane 1 = positive control, Lane 2 = 1 kb DNA ladder, Lane 3 = negative control, Lane 4–6 = transgenic line G2.H2-4, and Lane 7–9 = transgenic line G2.H3-5.

**Figure 4:**
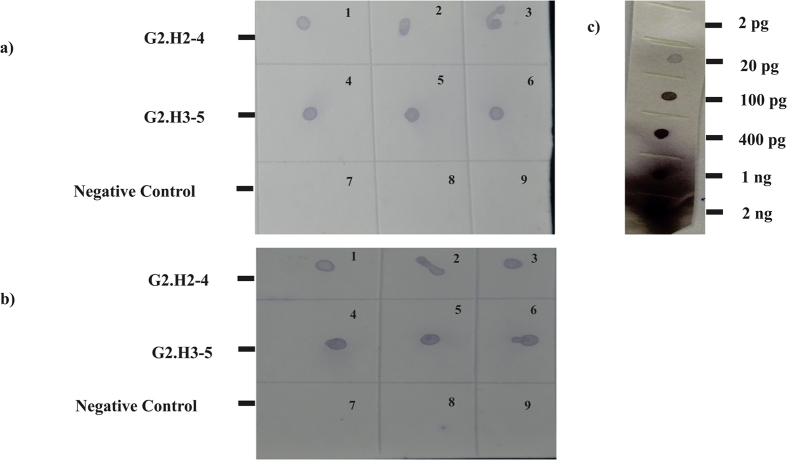
RNA dot blot showing relative expression of dsRNA in the transgenic tobacco lines (**a**) expressing the target gene *AChE*: Block 1–3 = transgenic line G2.H2-4, Block 4–6 = G2.H3-5, and Block 7–9 = negative control plants; (**b**) expressing the target gene *EcR*: Block 1–3 = transgenic line G2.H2-4, Block 4–6 = G2.H3–5 and Block 7–9 = negative control plants; and (**c**) serial dilution of known concentrations of the positive control RNA.

PCR positive transgenic tobacco lines G2.H3-5 and G2.H2-4 (Fig. 3) were subjected to real-time qPCR for gene expression analysis of *nptII* gene (Figure S2) and RNA dot blot assay for dsRNA/siRNA detection of whitefly target genes *AChE* and *EcR*. Total RNA was isolated from 3 plants of each transgenic line, and from three non-transgenic control plants. DIG-labelled RNA probes for the G2 construct, 200 bp in size for each gene fragment, were hybridized to the RNA spots on a nylon membrane (+Hybond, Amersham, UK). *In-vitro* transcription of cloned *AChE* fragment from a pTZ57R/T vector was used as a positive control and it was serially diluted to the known concentration of the transcribed RNA isolated from the transgenic tobacco lines. Color detection by NBT/BCIP revealed the presence of transcribed *AChE* and *EcR* fragments in the transgenic lines, and the accumulation was estimated using a known concentration of positive control RNA (Fig. 4).

### Real time PCR quantification of target gene expression

**Figure 5:**
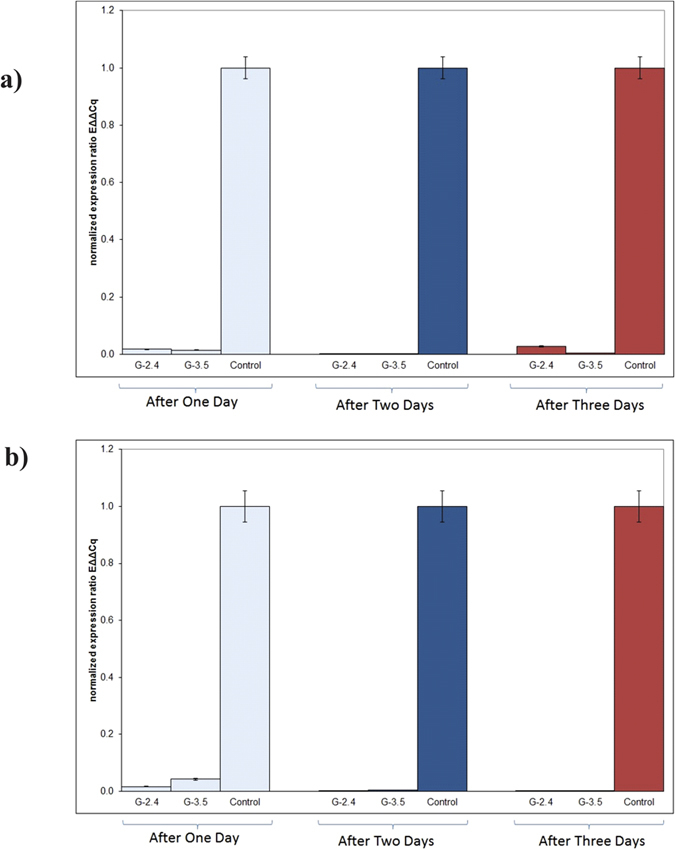
Results of the qPCR indicates the significant downregulation of the genes *Acetylcholinesterase (AChE*) and *Ecdysone Receptor (EcR*) in adult whitefly, and RT-qPCR results for *AChE* and (**a**) *EcR* (**b**).

Transient expression of dsRNA in tobacco lines G2.H2-4 and G2.H3-5 were selected for insect feeding bioassays in order to evaluate the dsRNA/siRNA mediated down regulation of the target genes (*AChE* and *EcR*). Real time qPCR was performed to assess the suppression of gene expression in *B. tabaci*. Whiteflies were allowed to feed on the transgenic tobacco plants producing dsRNA against *AChE* and *EcR* genes of the *B. tabaci.* The RT-qPCR results demonstrated highly significant suppression of the expression of both target genes. The RT-qPCR results showed significantly reduced expression of both whitefly target genes compared to whiteflies allowed feeding exposure on the negative control plants, for three consecutive days. Collection of whiteflies from transgenic and control plants at three different time points was carried out for down regulation assessment. The RT-qPCR analysis revealed approximately 98% reduction in *AChE* and *EcR* mRNA expression after 24 hrs in the transgenic lines, compared to the negative control plants, respectively (Fig. 5).

### Whitefly mortality following exposure to transgenic plants expressing dsRNA

**Figure 6:**
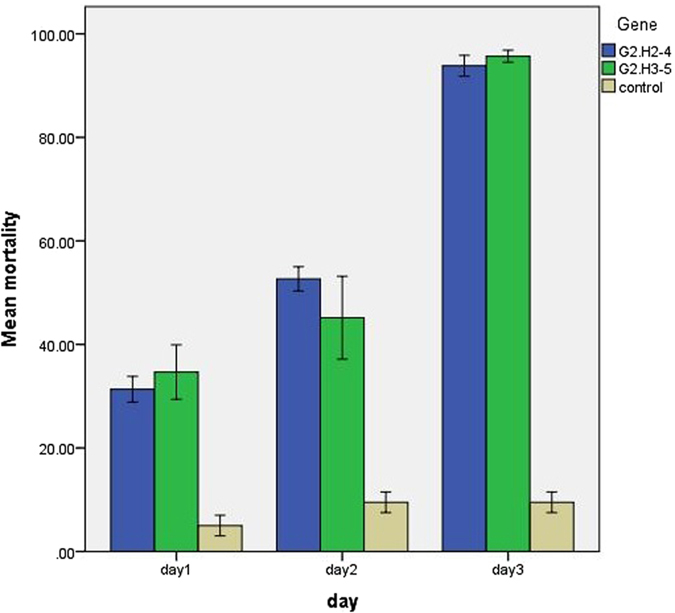
Mortality of whiteflies post-feeding exposure on transgenic tobacco lines, compared with control non-transgenic lines statistically analyzed and presented in a bar chart for the three-day bioassay (G2.H2-4 & G2.H3-5 represents two different transgenic lines).

Approximately, 200 whiteflies were allowed to feed on transgenic as well as control tobacco plants grouped in separate cages. More than 30% mortality was observed after one day of feeding exposure to the *AChE* and *EcR* transgenic lines, but no significant mortality was observed in whiteflies exposed to the negative control tobacco plants. At two days post-feeding exposure, whitefly mortality on transgenic tobacco plants was 40–45% for the transgenic lines G2.H2-4 and G2.H3-5. After three days post-feeding exposure, greater than 90% mortality was observed in both transgenic lines, while insignificant mortality was observed in negative control tobacco plants (Fig. 6).

## Discussion

The whitefly *B. tabaci* poses a threat to agricultural production worldwide. An alternative to chemical pesticides involves the development of whitefly resistance using plant breeding approaches, but successes have been minimal, while also being time and labor intensive^41^. Introduction of transgenic Bt cotton has helped farmers for more than two decades to control lepidopteran insect pests, resulting in decreased pesticide use and lowered management costs^42^. However, to the present Bt has not been effective against sap-sucking insects especially whitefly. Another solution involves the use of RNAi that can selectively target the genes belonging to particular insect species, thereby avoiding the targeting of beneficial insects. Over the past few years, this technology has been shown effective for insect gene silencing in functional genomics studies. The methods adopted for RNAi in insects including dsRNA spray, micro-injection and artificial diet based feeding were, although successful, are not yet suitable for field applications. However, the expression of dsRNAs in transgenic plants to silence target genes has been demonstrated to be economic for insect control at the field level^11^,^41^.

The whitefly *B. tabaci* is considered one of the most damaging among agricultural insect pests in tropical to temperate regions of the world and also a host of different mites^43^,^44^. Phylogenetic studies have identified several different endemic cryptic species in Pakistan that belong to the Asia I and II major clades, as well as the exotic MEAM I haplotype (B biotype), which has been widely established nearly globally in agricultural systems^45^. The analysis of *AChE* and *EcR* genes as prospective RNAi targets that result in mortality of *B. tabaci* demonstrated great promise for target design that takes into account conserved regions of genes that are likewise conserved across the *B. tabaci* sibling species group, and the greenhouse whitefly *T. vaporariorum*. Thus the results reported here illustrate the utility of targeting homologous regions of insect genes for RNAi, to achieve broad-spectrum whitefly control.

Pesticides, such as organophosphates, pyrethroids and carbamates have become less effective for whitefly control, and their use at high concentrations worsens environmental conditions and health of non-target organisms^28^. In this study, whiteflies exposed to tobacco plants transgenically expressing dsRNA showed significant down-regulation of the target genes, *AChE* and *EcR*, that lead to a high rate of mortality. This was accomplished by fusing the partial fragments of both the genes, separated by a 115 bp intron, expressed under control of the FMV promoter, which drives viral gene expression in various plant tissues and organs, including the phloem. Analysis by RNA dot blot analysis of the transgenic tobacco plants, compared with serial dilutions of the positive control, indicated that the dsRNA/siRNA relative concentration ranged from 10 to 100 pg. Moreover, down regulation of the target genes in the transgenic plants was corroborated in bioassays in which whiteflies given feeding exposure to tobacco plants, showed significantly higher mortality, compared to the non-transgenic control tobacco plants. Results from the RT-qPCR confirmed that early mortality was associated with the down-regulation of the *AChE* and *EcR* genes in whiteflies exposed to dsRNA expressing tobacco plants, and that whiteflies feeding on non-transgenic control plants showed significantly higher expression of the target genes, compared to exposed to transgenic lines expressing the respective genes. The results from these experiments confirmed the vital role of the two target genes (*AChE* and *EcR*) in life processes. Specifically, knock downs of *AChE* and *EcR* interfered with whitefly mating and oviposition before it resulted in mortality. The latter genes also are targets of insecticides such as Imidacloprid^46^ and benzoyl hydrazine^47^,^48^ that affect the neuronal and steroid functions of insects. Previously RNAi technology has been shown effective in down-regulating expression of midgut-expressed osmoregulatory genes, aquaporin and alpha glucosidase of whitefly^32^, for which greater than 70% mortality was shown, six days post-feeding on transgenic tobacco plants, compared to negative controls.

Distinct genetic and physiological differences are expected to occur among agriculturally important insect species, making important the careful selection of target gene(s) for each particular insect species, while also minimizing negative effects on beneficial or non-target insects that may be exposed to transgenic plants. Numerous considerations are thereby important for the achievement of safe and successful transgenic plant-mediated insect management. This study demonstrated the potential for dsRNA biopesticide technology using combination of genes in a single dsRNA construct, and opens up new avenues for gene pyramiding. Perhaps RNAi could also complement other approaches, such as Bt technology, to achieve broad-spectrum resistance against different groups of sucking and chewing insects.

## Materials and Methods

### Whitefly *B. tabaci* haplotype or species identification

Approximately twenty-five adult whiteflies were collected from cotton/agricultural fields in 14 districts/different locations of Pakistan where previous analyses have shown the predominance of *B. tabaci* species that belong to either with the Asia II major clade, and/or identified as the exotic B biotype^49^, or the greenhouse whitefly *Trialeurodes vaporariorum* (West).

Five adults were selected from each group of whitefly samples collected and were individually subjected to PCR amplification of the *mitochondrial cytochrome oxidase I (mtCOI*) gene was carried out using Phire Animal Tissue Direct PCR Kit (cat no. F140WH), according to the manufacturer’s instructions. In brief, adult whiteflies were homogenized in 20 μL dilution buffer, containing 0.5 μL DNA Release provided in the kit. The mixture was heated at 98 °C for 2 minutes, and centrifuged for 2 minutes at 13,000 rpm at room temperature. The DNA was stored at −20 °C after using 1 μL of supernatant in a 20 μL PCR reaction. The primers used for *mtCOI* amplification were: COI-3 F5′-TTGATTTTTTGGTCATCCAGAAGT-3′ and COI-3 R5′-TCCAATGCACTAATCTGCCATATTA-3′^50^.

The reaction contained 10 μL 2X Phire Animal Tissue PCR Buffer, 0.4 μL Phire Hot Start II DNA Polymerase, 0.25 μL forward and reverse primer each (10 μmol) and final volume made up with ddH_2_O. The cycling parameters were: 1 cycle for denaturation at 95 °C for 5 minutes, 34 cycles for 30 seconds at 95 °C, 30 seconds at 45 °C, 45 seconds at 72 °C and a final extension for 1 cycle at 72 °C for 10 minutes The PCR products of 900 bp in size were subjected to DNA sequencing (Genomics and Bioinformatics Research Unit, USDA-ARS, MS, USA).

Sequence aligned was carried out and a Maximum Likelihood (ML) phylogenetic tree was reconstructed using Kimura 2-parameter model with 1000 bootstrap value using MEGA 6.0. The sequence from the greenhouse *T. vaporariorum* was used as an outgroup (AF418672).

The raw sequences were assembled with SeqMan software of the DNASTAR package v 5.0, and were BLAST against the NCBI nr database. The *B. tabaci* colony used in bioassay experiments was established by placing 2 separate pairs of Asia II adults on tobacco plants and allowing them to feed and mate. The colony was thereafter maintained by periodic serial transfer to cotton plants in a greenhouse at NIBGE, Faisalabad, Pakistan.

### Total RNA isolation and cDNA synthesis from adult whitefly

Total RNA isolation from the collected samples of adult whiteflies was carried out using TRIzol reagent (Life Technologies cat no. 15596018), per the manufacturer’s recommendation. The homogenate of whitefly tissue samples was made with 500 μL of TRIzol reagent and incubated at room temperature (RT) for 5 minutes followed by centrifugation at 13000 rpm (at 4 °C) for 5 minutes. The supernatant was collected in separate nuclease free 1.5 mL eppendorf tubes and 250 μL of chloroform was added. The contents were mixed briefly and were incubated at room temperature for 5 minutes followed by centrifugation at 13,000 rpm (at 4 °C) for 5 minutes. The aqueous phase was transferred to another nuclease free 1.5 mL eppendorf tube and 500 μL of pre-chilled isopropanol was added and incubated at room temperature for 5 minutes followed by centrifugation at 13000 rpm (at 4 °C) for 5 minutes. The RNA pellet was washed with 75% ethanol, and dissolved in 20 μL nuclease free water. The cDNA was synthesized using 0.2 μg of RNA template from each whitefly samples using SuperScript™ IV First-Strand cDNA synthesis kit (Thermo Fisher Scientific, cat no. 18091200) following the manufacturer’s recommended protocol.

### Amplification and sequencing of whitefly *AChE* and *EcR* fragments

The cDNA from each sample was diluted to 1:9 and 1 μL of diluted cDNA was used in the PCR reaction using DreamTaq Green PCR Master mix 2x (Thermo Fisher Scientific, cat no. K1081). The PCR reaction contained 12.5 μL of Green PCR Master mix and 1 μL template cDNA with 0.5 μL each forward and reverse primers (10 μmol) added to a final volume of 25 μL using nuclease free H_2_O.

The PCR primers used were: AChEF5′-CGGAATTCCGCACAATATCACGCTCTTC-3′ and AChER5′-GGCTCGAGTCGATAGCCTCAGGGATCTG-3′ and EcRF5′-AAGAATTCATGAACATCCATCCCCAGAG-3′ and EcRR5′-AACTCGAGTCAGCATCATACCTCCTTGC-3′ for all the species and *T. vaporariorum* to amplify acetylcholinesterase and ecdysone receptor, respectively.

The PCR products were subjected to DNA sequencing (Scheffler lab, Genomics and Bioinformatics Research Unit, USDA-ARS, MS USA). The DNA sequence fragments corresponding to the *B. tabaci* and *T. vaporariorum* whitefly *AChE* and *EcR* gene fragments, respectively, were aligned using MegAlign software (DNASTAR package v 5.0) and the ClustalW algorithm. A pairwise distance matrix was computed for each gene in Sequence Demarcation Tool 1.2 (SDT) using the ClustalW method. The alignment and matrix were used to identify a 60 bp conserved region around which dsRNA could be designed.

### Cloning of *AChE* and *EcR* gene fragments in TRV-VIGS plasmid vector

The primer pairs, F5′-GTGAATTCCGCACAATATCACGCTCTTCG-3′ and R5′-TGGGATCCCGTGTGAGGGCAGCCGACAGCC-3′ and F5′-AAGAATTCATGAACATCCATCCCCAGAGG-3′ and R5′-ACGGATCCACTACATGCCTTTAATAAAGC-3′ were designed to amplify a 200 bp product corresponding to *AChE* and *EcR* gene fragments, respectively, with the addition of restriction sites for *Eco*RI and *Bam*HI to facilitate cloning. The PCR product was purified with 3M sodium acetate and double digested with *Eco*RI and *Bam*HI using 2x Tango buffer (Thermo Fisher Scientific). The TRV2 component of the TRV-VIGS vector was also digested using the same enzymes. The restricted plasmid vector and purified restricted PCR products were then ligated. The plants were infiltrated with TRV1+ [TRV2 + *ACHE*](*AChE* was cloned in TRV2 component) and TRV1 + [TRV2 + *EcR*](*EcR* was cloned in TRV2 component). The control plants were infiltrated with wild type TRV1 + TRV2.

### Agro-infiltration with TRV-VIGS containing dsRNA

The vector component the TRV2, containing gene of interest, and TRV1 were transformed in *Agrobacterium tumefaciens* strain GV3101. Positive colonies were confirmed by a modified method involving colony PCR^51^, and inoculated into 50 mL Luria Bertani medium with addition of 50 mg ml^−1^ kanamycin and 20 mg ml^−1^ rifampicin. The cultures were centrifuged after 48 hours of incubation at 28 °C and were resuspended in induction medium (10 mM 2-N-morpholino ethane sulfonic acid with pH = 5.5, 10 mM MgCl_2_ and 200 μM acetosyringone) and were held overnight at room temperature. The optical density (O.D.) was maintained at 2.0, and cells were suspended in 10 mM MES buffer, pH 5.5. The VIGS vector components, TRV1 and TRV2, were mixed 1:1 and used to infiltrate *N. tabacum* plant leaves 25-days after plants were transplanted. The tobacco plants were held in the dark for two days, and then placed in an environmentally controlled greenhouse, maintained as described above. Similarly, agro-inoculation of tobacco plants with the plasmid vector minus the dsRNA hairpins, were established separately using the approach described above.

### Construction of dsRNA expression cassette

The sequence of the whitefly *B. tabaci,* MEAM I haplotype (B biotype) genes used to design the dsRNA hairpin cassettes for *AChE* (GenBank accession number EF675187.1) and *EcR* (GenBank accession number EF174329.1) were retrieved from the NCBI database. These sequences were analyzed for homology with other available sequences in the nr database using the NCBI BLAST search tool, and specifically against the human genome. Sequence regions with a 0% BLAST hit to the human genome sequence were used for commercial synthesis (Life Technologies, Thermo Fisher Scientific, USA) of the fragments, joined in the sense (200 bp each) and antisense (200 bp each) orientation, using an intron of *A. thaliana* as a spacer between the sense and anti-sense fragments to yield a hairpin structure upon expression. Suitable restriction sites were introduced into the fragments to allow cloning into the plasmid vector, pJIT163. The resultant 931 bp fragment was cloned into the plasmid vector for expression under the control of a *Figwort mosaic virus* (FMV) promoter and CaMV35S terminator. The *SacI* and *HindIII* restriction sites were introduced and used to replace the 2xCaMV35S promoter of pJIT163 vector with the FMV promoter. The complete cassette [*SacI*-*acetylcholinesterase* (sense)-*ecdysone receptor* (sense)-Intron-*acetylcholinesterase* (anti sense)-*ecdysone receptor* (anti sense)-*EcoRV*] was cloned into the binary vector pCambia2300 (GSL Biotech LLC Chicago, USA) for tobacco plant transformation using restriction sites *SacI* and *EcoRV*. The *EcoRV* restriction site produced a blunt end cut and was used because of the limited number of options available for further cloning. The pCambia2300 vector was restricted using *SacI* and *SmaI* sites for compatible ligation because *SmaI* produced a blunt end compatible with the *EcoRV* restriction site.

### Transgenic tobacco plants

The cassette developed in pJIT163 vector initially, was restricted with restriction enzymes *Sac*I and *EcoR*V and ligated into the plant transformation binary vector pCambia2300. Using the binary plasmid vector pCambia2300 containing the cloned dsRNA cassette, the plasmid was transformed in the *A. tumefaciens* strain LBA4404 using electroporation, at 1.8 kV. Positive transformants were inoculated in 50 mL LB media for 48 h at 28 °C. The *N. tabacum* explants were agro-transformed^52^, with the gene constructs, and selected by direct organogenesis using established procedure and the transformants were selected on 500 mg/L of kanamycin. Transgenic plants were grown in a tissue culture room at 25 ± 2 °C. Plants were transplanted to plastic pots, and transferred to a greenhouse maintained under controlled conditions, at 25 ± 2 °C with 60–70% relative humidity. The insertion of T-DNA was confirmed by PCR (Fig. 3) using Dream Taq Green PCR Master Mix (2x) (Thermo Fisher scientific, USA). The cycling parameters of PCR were 94 °C for 3 minutes (1 cycle), 94 °C for 30 seconds, 54 °C for 30 seconds and 72 °C for 45 seconds (35 cycles) and a final extension step at 72 °C for 10 minutes. The primer pairs used for the confirmation were: G2F5′-CGCACAATATCACGCTCTTCGGC-3′ and G2R5′-ACTACATGCCTTTAATAAAGCTA-3′ for transgene confirmation while nptIIF5′-CTCACCTTGCTCCTGCCGAGA-3′ and nptIIR5′-CGCCTTGAGCCTGGCGAACAG-3′ for kanamycin resistance marker gene *nptII.*

### RNA dot blot analysis of dsRNA expression in transgenic tobacco

RNA was isolated from PCR positive transgenic lines according to the standard protocol by TRIzol reagent (Thermo Fisher scientific, USA). RNA concentrations of the samples were determined using a Nanodrop 2000 spectrophotometer (Thermo Fisher scientific, USA). Dilutions of the RNA samples were made by dissolving 15 μg of total RNA in 66% formamide, 21% formaldehyde and 13% MOPS buffer and incubating at 65 °C for 15 minutes for denaturation. RNA samples dissolved in dilution buffer were spotted onto positively charged nylon membrane (Hybond-N+, GE-Amersham, UK) soaked with 6x SSC solution (pH = 7) and dried on sterilized Whatman filter paper. Spots of RNA were then fixed under UV, cross linked with the energy output 1200 μjoules/cm^2^ per second for 30 seconds (twice) at 300 nm. DIG (Dioxigenin-Labelled UTP) labelled RNA probe of *AChE* and *EcR* were made using DIG RNA labelling kit, according to the manufacturer’s protocol (Sigma-Aldrich). The probe was denatured at 65 °C for 5 minutes before hybridization onto a nylon membrane. After washing and blocking, RNA spots were detected by the chromogenic method, with a 6-hr incubation in NBT/BCIP at room temperature.

### Whitefly mortality on transgenic tobacco

Adults of the *B. tabaci* (Asia II I) were collected from cotton plants grown in the greenhouse, at 60–70% relative humidity, 26 ± 2 °C, and 16:8 h day/night photoperiod. Adults were transferred to and allowed to feed and oviposit on 70-day old tobacco plants grown maintained under the conditions described above.

Newly emerged adult whiteflies were released either on TRV-VIGS infiltrated, or transgenic tobacco plants, and the respective negative control tobacco plants, and allowed feeding exposure to the plants. Plants were monitored for ten continuous days in two replicated experiments for TRV-VIGS. Mortality was also recorded on daily basis for three consecutive days in case of transgenic tobacco plant bioassays (as insects were only able to survive for three days on transgenic tobacco plants). The mean percent mortality was calculated, and subjected to an analysis of variance (ANOVA) using the statistical software Graph Pad Prism 5 and SPSS version 20 for transient and transgenic experiments respectively. Tukey’s post-test of significance was performed for both bioassays to assess pairwise differences, and a *p*-value < 0.05 was considered significant.

Adult whiteflies were collected from transgenic plants at 1, 2 and 3 days after their initial release on transgenic tobacco plants. Total RNA was isolated from whiteflies using TRIzol reagent, and then treated with DNase I, according to the manufacturer’s instructions (Thermo Fisher Scientific, USA). A Nanodrop 2000 spectrophotometer (Thermo Fisher scientific, USA) was used to quantify whitefly total RNA. From each RNA sample, 0.5 μg was used for cDNA synthesis, according to manufacturer’s protocol (Thermo Fisher Scientific, USA).

### Real time quantitative PCR analysis

Expression of the target genes, *AChE* and *EcR,* in adult *B. tabaci* was quantified by RT-qPCR. The 25 μl reaction mixture contained 12.5 μl SYBR^®^ Green, 0.25 μl each of the forward and reverse primers (0.1 pmole), 2.5 μl cDNA (~25 ng) and 9.5 μl water. Primer sequences: qAChEF5′-GGAGGAGGGCAACTACTGGAT-3′ and qAChER5′-CACCGCCTGGATGAAACTG-3′ for *AChE* gene, qEcRF5′-GTTTGCAACTAACCAGCCGTATA-3′ and qEcRR5′-GGAGCAGATCCTCCACAGTATC-3′ for *EcR* gene while q18SF5′-GACCGGAGCTTGCAATTGTTC-3′ and q18SR5′-ATCGCCGCGAGGTTATGAC-3′ were used for 18S ribosomal RNA gene as internal control. The PCR reaction parameters for both primer pair combinations were: 1 cycle at 94 °C for 10 minutes followed by 40 cycles, at 94 °C (for 30 seconds), 53 °C (for 30 seconds) and 72 °C (for 30 seconds). The RT-qPCR reactions were run in triplicate, in a 96 well microtiter plate using Bio-Rad iQ5 thermal cycler (Bio-Rad, USA).

To assess the specificity of the amplification product, at the end of each run, a melt curve analysis was performed, from 60 to 95 °C, with an increment of 0.5 °C every 10 seconds Quantification results were analyzed using 2 −ΔΔCT method^53^. The 18 S ribosomal RNA (rRNA) gene was used to normalize the corresponding Ct values. Transcript levels were measured in whiteflies fed on transgenic lines and/or control plants and the fold change in the expression levels of *AChE* and *EcR* was determined as described, above.

## Additional Information

**How to cite this article**: Malik, H. J. *et al*. RNAi-mediated mortality of the whitefly through transgenic expression of double-stranded RNA homologous to acetylcholinesterase and ecdysone receptor in tobacco plants. *Sci. Rep.* **6**, 38469; doi: 10.1038/srep38469 (2016).

**Publisher’s note:** Springer Nature remains neutral with regard to jurisdictional claims in published maps and institutional affiliations.

## Supplementary Material

Supplementary Information
