# Supplementary material for: RNAi-mediated mortality of the whitefly through transgenic expression of double-stranded RNA homologous to acetylcholinesterase and ecdysone receptor in tobacco plants
Source: Sci Rep. 2016 Dec 8;6:38469. doi: 10.1038/srep38469 (PMC5143975; doi:10.1038/srep38469)
Supplement: Supplementary Information [file srep38469-s1.pdf]

-Supplementary information-

RNAi-mediated mortality of the whitefly through transgenic expression of double-stranded RNA homologous to acetylcholinesterase and ecdysone receptor in tobacco plants

Hassan Jamil Malik[1,2], **Amir Raza**[1,2], **Imran Amin**[1], **Jodi A. Scheffler**[3], **Brian E. Scheffler**[4], **Judith K. Brown**[5] and **Shahid Mansoor**[1*]

[1]Molecular Virology and Gene Silencing Laboratory, Agricultural Biotechnology Division, National Institute for Biotechnology and Genetic Engineering (NIBGE), Jhang Road, PO Box # 577, Faisalabad, Pakistan

[2]Pakistan Institute of Engineering and Applied Sciences (PIEAS), Islamabad, Pakistan

[3]USDA-ARS, Crop Genetics Research Unit, 141 Experiment Station Rd, Stoneville, MS USA 38776

[4]USDA-ARS, Genomics and Bioinformatics Research Unit, 141 Experiment Station Rd, Stoneville, MS USA 38776

[5]School of Plant Sciences, The University of Arizona, Tucson, AZ 85721, USA

***Corresponding author: Shahid Mansoor**

Agricultural Biotechnology Division, National Institute for Biotechnology and Genetic Engineering (NIBGE), Jhang road, Faisalabad, Pakistan,

Phone #: +92-41-9201471

Fax #: +92-41-6201472

shahidmansoor7@gmail.com

Supplementary information:

The tree was re-constructed by using maximum likelihood (ML) (Fig. S1). The mtCOI reference sequences were obtained from GenBank for *B. tabaci* haplotypes of Asia II, I, and MEAM I (B biotype), and the greenhouse whitefly, *T. vaporariorum,* which was used as outgroup for the phylogenetic analysis. The GenBank sequence number and their references in the literature are shown in Table S1. *T. vaporariorum* was identified from two districts of the province Khyber Pakhtunkhwa (KPK) of Pakistan (authors, unpublished). Also, Asia II I was identified in the Punjab Province of Pakistan while the MEAM I (B biotype) was found to occur in the coastal region of the Sindh Province. The sequence distance matrix report was computed using Sequence Demarcation Tool (SDT) v1.2.Results indicated that *T. vaporariorum* (AF418672) shared only ±72 % nucleotide identity with the species of *B. tabaci*, whereas, the Asia II-1 and the MEAM I shared 100 % nucleotide identity.

The real time quantitative PCR was performed for the assessment of transgene expression in transgenic tobacco plants (Fig. S2). Two primer pairs including the *nptII* gene as well as gene specific primers were used for this purpose. The RT-qPCR results showed higher expression level in case of *nptII* gene whereas only a very low level of expression was detected in case of the gene of interest. The higher level *nptII* expression indicates that the transgene has been successfully integrated into the plant genome. The possible reason for very low expression of gene of interest is that the messenger RNA being produced immediately gets converted to double stranded-RNA which will be quickly processed into short interfering RNAs. A second observation was that the amount of mRNA total RNA preparations appeared to be low, and was perhaps due to its propensity for degradation during isolation and DNase treatment. Often, RNA dot blot has been used for short RNA detection and confirmation of transgene expression particularly when the expected product is dsRNA.

Figure legend S1: Maximum likelihood tree based mtCOI analysis of whitefly *B. tabaci* haplotypes/cryptic species identified in selected field collections from Pakistan.

Figure legend S2:

Results of the real time qPCR from transgenic tobacco plants where, **(a)** represents the relative gene expression of *nptII* gene in the transgenic lines G2.H2-4 and G2.H3-5 normalized with the 18S rRNA gene of *N. tabacum.* Non-transgenic tobacco plants were used as control also mentioned as wild-type (*wt*) where no *nptII* expression was detected thereby confirming the integration of transgene only in transgenic plants. **(b)** Amplification products from RT-qPCR displayed on 1% agarose gel where, L1-L3 represents the amplification of 18S rRNA gene from transgenic line G2.H2-4, L4-6 shows 18S rRNA gene amplification from transgenic line G2.H3-5, L7-9 demonstrates the 18S rRNA gene amplification from non-transgenic tobacco plants, L10 presents the 100 bp marker (Promega, USA), L11-L13 represents the product of *nptII* gene amplification in transgenic line G2.H2-4,L14-16 presents the *nptII* gene amplification in transgenic line G2.H3-5 and L17-19 demonstrates the absence of *nptII* gene in non-transgenic tobacco plants (*wt*).

Supplementary Table

Table S1: GenBank accessions of sequences used for whitefly *B. tabaci* and *T. vaporariorum* identification.

MtCOI Accessions	AChE	EcR	Whitefly species
KX675899	KU307111	KU307151	*Trialuerodes vaporariorum*
KX675900	KU307112	KU307152	*T. vaporariorum*
KX675901	KU307113	KU307153	*T. vaporariorum*
KX675902	KU307114	KU307154	*T. vaporariorum*
KX675903	KU307115	KU307155	*T. vaporariorum*
KX675904	KU307116	KU307156	*Bemisia tabaci*-MEAM1
KX675905	KU307117	KU307157	*B. tabaci*-MEAM1
KX675906	KU307118	KU307158	*B. tabaci*-MEAM1
KX675907	KU307119	KU307159	*B. tabaci*-MEAM1
KX675908	KU307120	KU307160	*B. tabaci*-MEAM1
KX675909	KU307121	KU307161	*B. tabaci*-MEAM1
KX675910	KU307122	KU307162	*B. tabaci*-Asia II I
KX675911	KU307123	KU307163	*B. tabaci*-Asia II I
KX675912	KU307124	KU307164	*B. tabaci*-Asia II I
KX675913	KU307125	KU307165	*B. tabaci*-Asia II I
KX675914	KU307126	KU307166	*B. tabaci*-Asia II I
KX675915	KU307127	KU307167	*B. tabaci*-Asia II I
KX675916	KU307128	KU307168	*B. tabaci*-Asia II I
KX675917	KU307129	KU307169	*B. tabaci*-Asia II I
KX675918	KU307130	KU307170	*B. tabaci*-Asia II I

Figure S1:

Cytochrome oxidase I

COI accessions	Species/Biotypes
KX675899	*T.vaporariorum*
KX675900	*T.vaporariorum*
KX675901	*T.vaporariorum*
KX675902	*T.vaporariorum*
KX675903	*T.vaporariorum*
KX675904	*B. tabaci*-MEAM1
KX675905	*B. tabaci*-MEAM1
KX675906	*B. tabaci*-MEAM1
KX675907	*B. tabaci*-MEAM1
KX675908	*B. tabaci*-MEAM1
KX675909	*B. tabaci*-Asia II I
KX675910	*B. tabaci*-Asia II I
KX675911	*B. tabaci*-Asia II I
KX675912	*B. tabaci*-Asia II I
KX675913	*B. tabaci*-Asia II I
KX675914	*B. tabaci*-Asia II I
KX675915	*B. tabaci*-Asia II I
KX675916	*B. tabaci*-Asia II I
KX675917	*B. tabaci*-Asia II I
KX675918	*B. tabaci*-Asia II I
**AF418672**	*T. vaporariorum*
**FN821808**	*B. tabaci*-B/MEAM1
**JX993192**	*B. tabaci*-B/MEAM1
**AJ510065**	*B. tabaci*-Asia II I
**KR110175**	*B. tabaci*-Asia II I

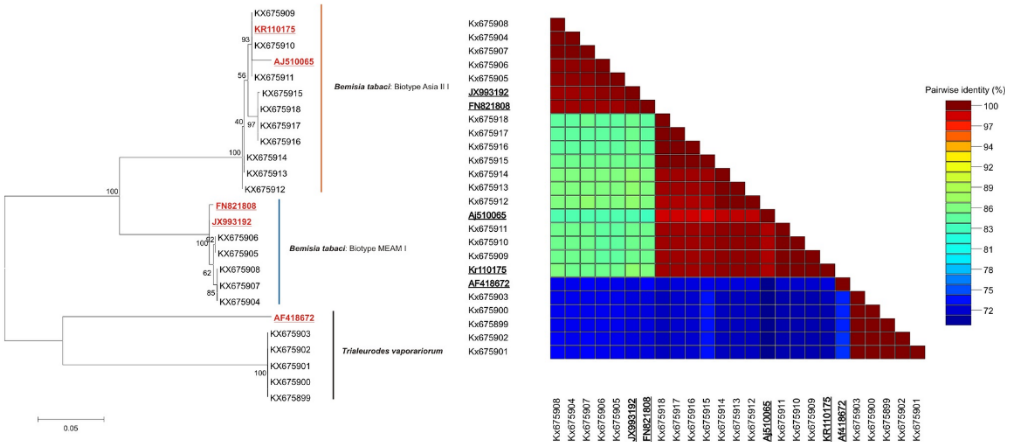

Figure S2:

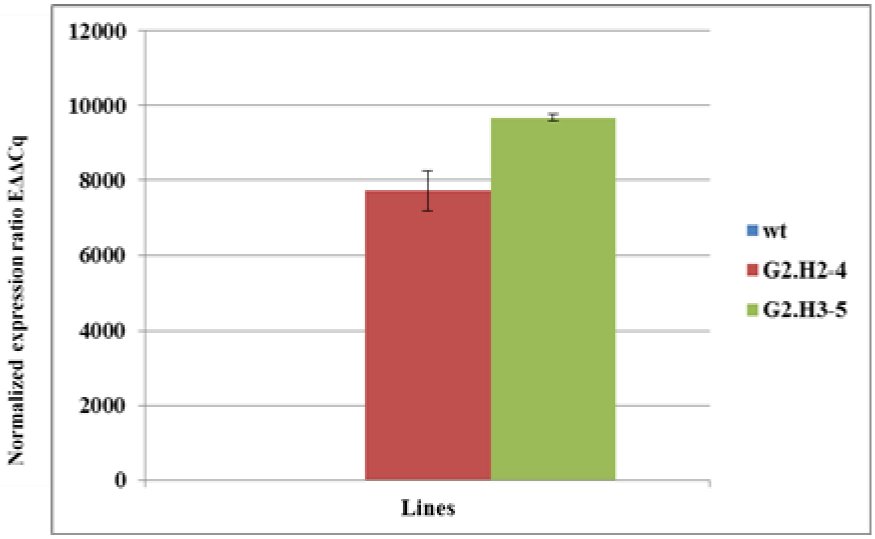

(a)

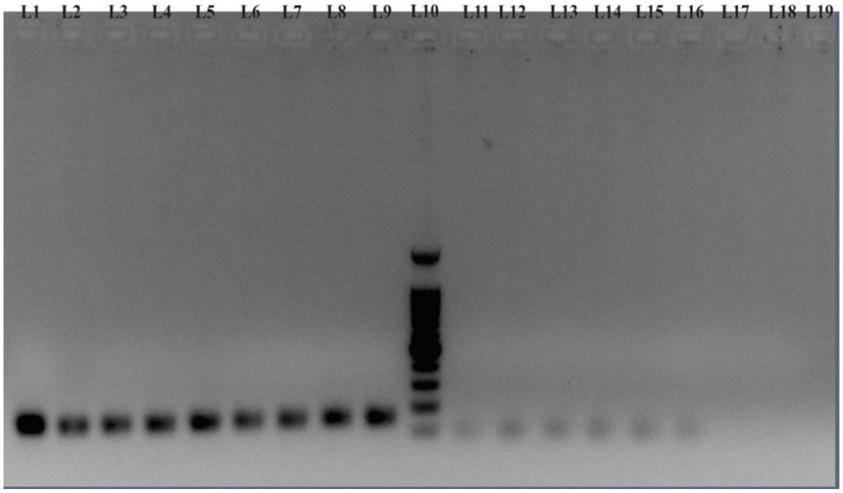

(b)
